# MEMS Device for Quantitative In Situ Mechanical Testing in Electron Microscope

**DOI:** 10.3390/mi8020031

**Published:** 2017-01-24

**Authors:** Xiaodong Wang, Shengcheng Mao, Jianfei Zhang, Zhipeng Li, Qingsong Deng, Jin Ning, Xudong Yang, Li Wang, Yuan Ji, Xiaochen Li, Yinong Liu, Ze Zhang, Xiaodong Han

**Affiliations:** 1 Beijing Key Lab of Microstructure and Property of Advanced Materials, Beijing University of Technology, Beijing 100124, China; wwxiaodongfy@emails.bjut.edu.cn (X.W.); zhangjf@emails.bjut.edu.cn (J.Z.); b201526001@emails.bjut.edu.cn (Z.L.); qsdeng@bjut.edu.cn (Q.D.); liw@bjut.edu.cn (L.W.); jiyuan@bjut.edu.cn (Y.J.); xcli@emails.bjut.edu.cn (X.L.); zezhang@zju.edu.cn (Z.Z.); 2 Department of Fundamental Sciences, Chinese People’s Armed Police Force Academy, Langfang 065000, China; 3 Research Center of Engineering for Semiconductor Integrated Technology, Institute of Semiconductors, Chinese Academy of Sciences, Beijing 100083, China; ningjin@semi.ac.cn; 4 College of Electronic Information and Control Engineering, Beijing University of Technology, Beijing 100124, China; yangliuge@bjut.edu.cn; 5 School of Mechanical and Chemical Engineering, The University of Western Australia, Crawley 6009, WA, Australia; 6 State Key Laboratory of Silicon Materials and Department of Materials Science and Engineering, Zhejiang University, Hangzhou 310008, China

**Keywords:** piezoresistive sensor, electron microscope, in situ mechanical test

## Abstract

In this work, we designed a micro-electromechanical systems (MEMS) device that allows simultaneous direct measurement of mechanical properties during deformation under external stress and characterization of the evolution of nanomaterial microstructure within a transmission electron microscope. This MEMS device makes it easy to establish the correlation between microstructure and mechanical properties of nanomaterials. The device uses piezoresistive sensors to measure the force and displacement of nanomaterials qualitatively, e.g., in wire and thin plate forms. The device has a theoretical displacement resolution of 0.19 nm and a force resolution of 2.1 μN. The device has a theoretical displacement range limit of 5.47 μm and a load range limit of 55.0 mN.

## 1. Introduction

Mechanical properties of materials are influenced strongly by their microstructures. Materials often show special mechanical properties compared to their bulk counterparts when the grain size or physical dimension is reduced to the nanometer scale [1,2,3,4,5,6,7,8]. For example, brittle Si and SiC show super-plasticity when their sizes are reduced to below ~100 nm [9,10,11,12,13]; the elasticity of nanosized copper can approach the theoretical elastic strain limit [14]; and the martensitic transformation can be completely suppressed in NiTi shape memory alloys when the sample thickness is below ~50 nm [15,16]. In this regard, it is important to develop capabilities to allow quantitative studies of the structure–property correlations of materials at the nano and atomic scales.

Many techniques and devices, adapted to scanning electron microscopes (SEM) or transmission electron microscopes (TEM), have been designed to allow in situ studies of structure–property relationships at the nano and atomic scales [12,17,18,19,20,21,22,23,24,25,26]. Some devices allow the observation of the microstructural evolution and simultaneous measurement of stress–strain curves, thus granting us an opportunity to understand the microscopic mechanisms of deformation and to better guide the development of new materials [27,28]. Techniques currently available for mechanical testing at nanoscale include nano indentation [29], bugling [30], resonance [31], bending [32], and micro-tensile testing [33] using micro/nanosized samples in SEM, TEM, and atomic force microscope (AFM). For TEM in situ analysis, one of the main functional requirements of the technique for structure–property correlation studies is to measure quantitatively, the stress–strain behavior of the sample in TEM whilst having negligible impact on the double-axis tilt of the TEM sample holder. Nano indentation has been adapted in TEM for in situ deformation measurement [34]. Nano indentation has high displacement and force resolutions of 0.03 nm and 0.1 μN [35], respectively, which is commonly used to determine elastic modulus, hardness, and stiffness of the material surface [36,37,38,39,40,41]. However, this technique places the displacement and force sensors at the end of the TEM holder, thereby prohibiting the β-axis tilt and partially limiting the orientation capability of the TEM.

Recently, MEMS-based devices, integrating actuators, sensors, and signal processing circuits, on a millimeter and even micrometer scales, have been developed at the head of the TEM holder for studying the structure–property relationships at the atomic scale [42,43,44]. Thus, the evolution of mechanical parameters and microstructure of materials have been simultaneously obtained. This has granted an opportunity to study directly the influence of microstructure on the mechanical properties of materials and has provided experimental evidences for designing new high performance materials. To measure accurately, the mechanical properties of nanosized materials, the displacement and force sensor resolutions need to be in the order of tens of nanometers and micronewtons, respectively. Displacement and force in electron microscopes can be determined mainly by imaging and capacitance. The imaging method determines the displacement by measuring the displacement difference between two flexible beams in TEM/SEM. The load is then given as the product of the force sensor beam displacement and beam spring constant [45,46]. By using the imaging method, it is not possible to output the stress–strain curve of the specimen in real time, thus restricting the deformation of materials to occur only at a very low strain rate. In the capacitance method, the displacement and force are measured based on the capacitance variance resulting from the deformation of the sensors located at the roots of the beams. This method has been widely used in harsh environments because of its superior properties, such as small temperature drift, low power consumption, good process compatibility, and direct signal output [47]. Differential capacitance based sensors have high force and displacement resolutions [27,48,49]. However, they have comparatively larger sizes, and thus, they can be used only in single-tilt or small angle double-tilt TEM holders, and they are difficult to use for atomic scale microstructural analysis.

Piezoresistive sensors fabricated with semiconductor materials have advantages such as easy fabrication, small size, and high sensitivity [50], and thus, they are good candidates for double-tilt TEM holders. The operating principle of piezoresistive sensors is that their resistance varies with external stress/strain. The electric signals of the resistance can be directly read out using a Wheatstone bridge circuit, which is small and can be integrated with the sensor using the MEMS technique. Because of its small size, a piezoresistive sensor can be easily placed on simple structures such as cantilever and clamped beams [49]. To improve their sensitivity, piezoresistors, in many cases, are fabricated on the beam surface perpendicular to the force direction. The force resolution of piezoresistive sensors has been reported to be as small as nanonewtons [51], thereby giving rise to the possibility of measuring the mechanical properties of nanosized materials. However, no MEMS-based devices with piezoresistive sensors seem to have been developed for structure–property studies at the nano/atomic scale.

In this work, we designed a piezoresistive sensor based MEMS device for mechanical deformation of materials with displacement and force resolutions of 7 nm and 2.2 μN, respectively. The device is small and has a potential to be adapted in SEM/TEM for quantitative uniaxial tensile testing of samples with thickness smaller than 100 nm and width of hundreds of nanometers. With this device in TEM/SEM, we can simultaneously study the mechanical properties and evolution of the material microstructure, which provides an opportunity to bridge the mechanical property-microstructure relationship from the micro to the atomic scales. An aluminum thin film sample, with a thickness of 510 nm, was subjected to trial test the device in SEM to validate its effectiveness.

## 2. Mechanical Testing System

### 2.1. Description of the System

**Figure 1:**
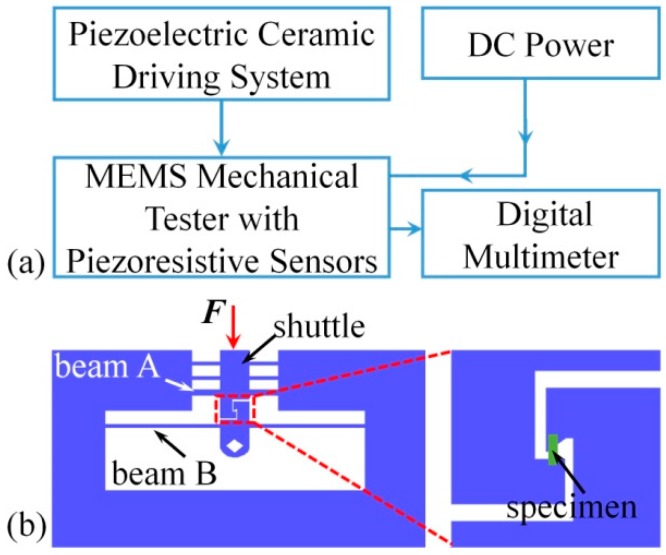
Schematic of the testing system in scanning electron microscopes (SEM): (**a**) the actuation control flow chart; and (**b**) design of the testing apparatus.

Figure 1 shows a schematic of the testing system design. Its operation control is given in Figure 1a. The system comprises an actuation system, a MEMS device, two single-system-power suppliers, and two digital multimeters (6½ digits). The two single-system-power suppliers provide a DC operating voltage for the two piezoresistive sensors. The beams on the device are driven by a piezoceramic actuator with a travel distance of 100 μm and a minimum step of 7 nm. Two digital multimeters were used to collect the sensor output voltages.

The MEMS device was fabricated using bulk silicon microfabrication process, and the design is illustrated in Figure 1b. Two sensors, A and B, were placed on two beams, A and B, to measure the displacement and force. Sensor A was placed at the root of beam A to measure the deflection of its center. To maintain the stability of sensor A, two more clamped beams parallel to beam A were connected with it by a shuttle, as can be seen in Figure 1b. Sensor B was placed at the root of beam B, which is longer than beam A, to measure the deflection of beam B. The deformation strain of the specimen is calculated using Equation (1):

(1) $$\varepsilon =\frac{x_{a}-x_{b}}{l_{0}}$$

where *x*_a_ and *x*_b_ are the center deflections of beams A and B, respectively, and *l*_0_ is the initial length of the specimen.

The load applied on the specimen approximately equals the driving force acting on the beam center, which was calculated based on the deflection of beam B. The stress is given as follows:

(2) $$\sigma_{s}=\frac{F_{b}}{S}$$

where *F*_b_ is the driving force acting on beam B and *S* is the cross-sectional area of the specimen. Using a push-to-pull structure, the external compressive stress on the specimen was transferred to a tensile stress, as shown in Figure 1b.

### 2.2. Sensor Design

**Figure 2:**
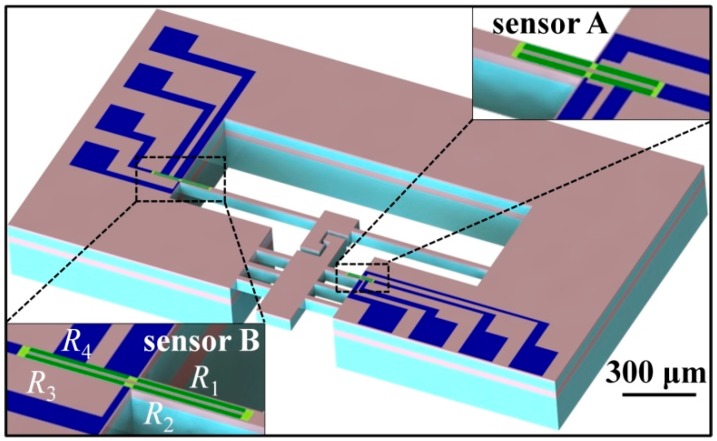
Sketch of the micro-electromechanical systems (MEMS) device with piezoresistive sensors.

Figure 2 shows the MEMS device with two piezoresistive sensors. The overall dimensions of the device are 1.3 mm × 2.4 mm × 0.44 mm, thus they can be easily installed on the sample stage at the head of a TEM hold. Four piezoresistors indicated by green were prepared on beams A and B, as shown in the insets of Figure 2. The four sensors form a half Wheatstone bridge, to allow a precise measurement of the resistive variance. The two piezoresistors, *R*_1_ and *R*_2_, located at the roots of the beams A and B, measure the beam deflection. The resistances of the other two resistors, *R*_3_ and *R*_4_, attached to the substrate, remain constant during the beam deflection. The four resistors are electrically connected with aluminum (Al) interconnects. To improve the consistency of the process and to partially compensate the influence of temperature, the resistors on the beam and those on the substrate were placed as close as possible to each other [52]. The beams and the piezoresistors are aligned along the <110> direction on the (100) plane of a silicon wafer substrate for obtaining a better sensor sensitivity [53].

**Figure 3:**
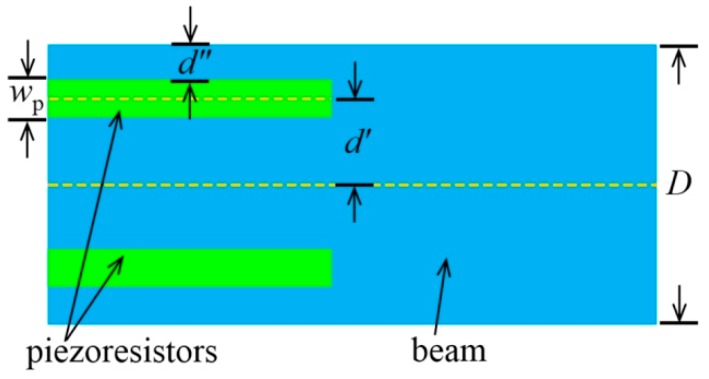
Schematic of the piezoresistor beam positions and the parameters used in Equations (4) and (5).

When the clamped beams deform upon an in-plane force, stress will concentrate at the beam roots. The stress is free at the neutral plane of the beam and increases gradually with increasing distance from the neutral plane. The variance of the resistance is given in Equation (3) [54]:

(3) $$\frac{\Delta R}{R}=\pi_{l}\sigma_{l}+\pi_{t}\sigma_{t}$$

where σ_l_ and σ_t_ are the longitudinal and transverse stresses, and π_l_ and π_t_ are the piezoresistance coefficients along the longitudinal and transverse directions. Since σ_t_ is much smaller than σ_l_, the term $\pi_{t}\sigma_{t}$ can be neglected [51]. Figure 3 shows the positions of the piezoresistors on the beam and the parameters used in Equations (4) and (5). In the case of small deformation, the variance of the resistance can be written as:

(4) $$\frac{\Delta R}{R}=\frac{6\pi_{l}Ed^{\prime }\omega }{L^{3}}(L-l_{p})$$

where *E* = 169 GPa [55] is the Young’s modulus of silicon in <110> direction, *L* is the beam length, *l*_p_ is the piezoresistor length, ω is the beam center deflection, and *d*′ is the distance between the resistor centerline and neutral plane of the beam. The value *d*′ can be expressed as:

(5) $$d^{\prime }=\frac{D}{2}-\frac{w_{p}}{2}-d^{\prime \prime }$$

where *D* is the beam thickness (along the force direction), *w*_p_ is the width of the piezoresistor, and *d*″ is the distance between the outer edge of the piezoresistor and the beam. Figure 3 shows all the parameters in Equation (5).

When a tensile stress is applied to the specimen, assuming that the piezoresistors *R*_3_ and *R*_4_ have an equal resistance variance Δ*R*, the bridge output voltage is given as:

(6) $$V_{\mathrm{out}}\approx \frac{\Delta R}{2R}V_{B}$$

where *V*_B_ is the bridge bias voltage.

The displacement sensitivity ($S_{D}$) of the piezoresistive sensors is defined as the ratio of the output voltage to the beam deflection, and is given by combining Equations (4) and (6):

(7) $$S_{D}=\frac{3\pi_{l}V_{B}Ed^{\prime }}{L^{3}}(L-l_{p})$$

The piezoresistive sensor noise arises from both intrinsic and extrinsic sources. Johnson noise and 1/*f* noise are the two main intrinsic noises, and their noise power spectral densities are given in Equations (8) and (9), respectively [56]:

(8) $${{\bar{V}}_{J}}^{2}=\frac{4k_{B}Tl_{p}}{N_{p}q\mu_{p}d_{p}w_{p}}(f_{\max}-f_{\min})$$

(9) $${{\bar{V}}_{H}}^{2}=\frac{\alpha {V_{B}}^{2}}{N_{p}l_{p}d_{p}w_{p}}\ln\frac{f_{\max}}{f_{\min}}$$

where *k*_B_ is the Boltzmann constant, *T* is the absolute temperature, *N*_p_ is the dopant concentration, *q* is the amount of the carrier charge, μ_p_ is the hole mobility, *d*_p_ is the piezoresistor thickness, α is a non-dimensional fitting parameter depending on the annealing conditions, and *f*_max_ and *f*_min_ are the upper and lower measurement frequency limits. The displacement resolution of the sensors is defined as the ratio of the noise to the displacement sensitivity (only considering Johnson and 1/*f* noise), and can be written as:

(10) $$R_{D}=\frac{L^{3}\sqrt{\frac{4k_{B}Tl_{p}}{N_{p}q\mu_{p}d_{p}w_{p}}(f_{\max}-f_{\min})+\frac{\alpha {V_{B}}^{2}}{N_{p}l_{p}d_{p}w_{p}}\ln\frac{f_{\max}}{f_{\min}}}}{3\pi_{l}V_{B}Ed^{\prime }(L-l_{p})}$$

Optimized design can effectively decrease the noise power and improve the displacement resolution, i.e., the smallest signal that can be detected. According to Equation (10), the density of the noise power decreases with increasing piezoresistor width (*w*_p_). For a nearly constant *d″*, depending on the MEMS technology, the *d*′ will become shorter when *w*_p_ is increased, leading to a decrease in the displacement sensitivity. To obtain an optimal width (*w*_p-optimal_), the resolution *R*_D_ can be partially differentiated against the piezoresistor width. The optimal width thus obtained is:

(11) $$w_{p-\mathrm{optimal}}=\frac{D}{3}-\frac{2}{3}d^{\prime \prime }$$

**Figure 4:**
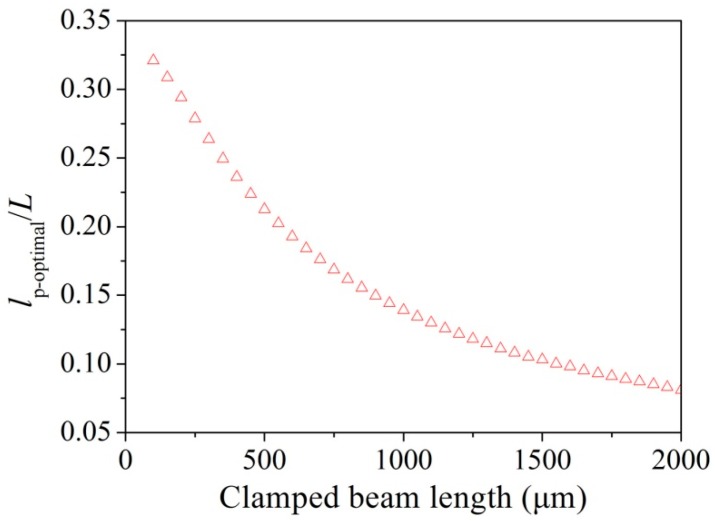
Evolution of the *l*_p-optimal_/*L* as a function of the clamped beam length (*L*).

According to Equations (8) and (9), increase in the length, *l*_p_, of the piezoresistor has an opposite influence on the power density of the Hooge and Johnson noises, i.e., a decrease in the Hooge noise but an increase in the Johnson noise. It is further noted from Equation (7) that increase in *l*_p_ can also lead to a loss in the displacement sensitivity. Because of the complicated effect of *l*_p_ on the displacement resolution and sensitivity, the resolution was partially differentiated against *l*_p_ to obtain an optimal length contributing to a high resolution. For each clamped beam length, *L*, an optimal *l*_p-optimal_ is obtained. Figure 4 shows the evolution of *l*_p-optimal_/*L* as a function of the clamped beam length. The results show that the *l*_p-optimal_/*L* decreases with increasing *L*.

Setting one of the supporting points to be an original point, the deflection/displacement of a position at a distance *x* from the original point can be written as Equation (12) according to mechanical analysis [57]:

(12) $$\omega (x)=\frac{Fx^{2}}{2EWD^{3}}(3L-2x)$$

where *W* is the beam width, and *F* is the force applied on the beam center. The longitudinal stress of each point can be expressed as [57]:

(13) $$\sigma (x,d^{\prime })=Ed^{\prime }\frac{d^{2}[\omega (x)]}{dx^{2}}$$

Substituting Equations (12) into (13), we obtain Equation (14):

(14) $$\sigma (x,d^{\prime })=\frac{3Fd^{\prime }}{WD^{3}}(L-2x)$$

According to Equation (14), the maximum longitudinal stress is located on the beam surface ($d^{\prime }=D/2$), roots (*x* = 0), and center (*x* = *L*), which can be written as:

(15) $$\left|\sigma_{l-\max}\right|=\frac{3FL}{2WD^{2}}$$

The sensors will fail when $\sigma_{l-\max}$ equals to the bending strength (3.70 GPa, [3]) of (100) single crystal silicon. According to Equation (15), the theoretical maximum forces that can be applied on the beam center and measured by the two sensors are calculated to be 888 mN for sensor A and 55.0 mN for sensor B, as listed in Table 1. According to Equation (12), the beam center deflection is given by:

(16) $$\omega =\frac{FL^{3}}{2EWD^{3}}$$

Then, the maximum displacements of the two sensors are calculated to be 5.47 μm for sensor A and 175.14 μm for sensor B, as listed in Table 1. Therefore, the theoretical range limits of load and displacement that can be measured by the MEMS device are 55.0 mN and 5.47 μm, respectively.

## 3. Experiment

### 3.1. Fabrication Process

**Figure 5:**
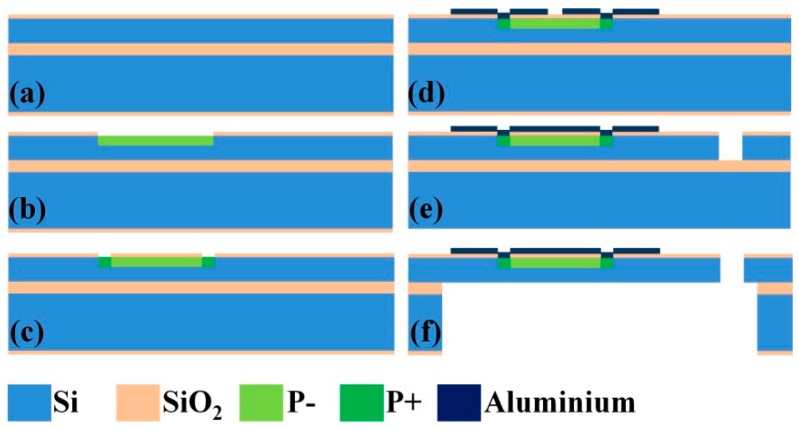
Schematic of the main steps to fabricate the MEMS device: (**a**) thermal oxidation; (**b**) p− doped; (**c**) p+ doped and contact hole; (**d**) interconnects and pads; (**e**) front side inductively coupled plasma (ICP); and (**f**) structure release.

**Figure 6:**
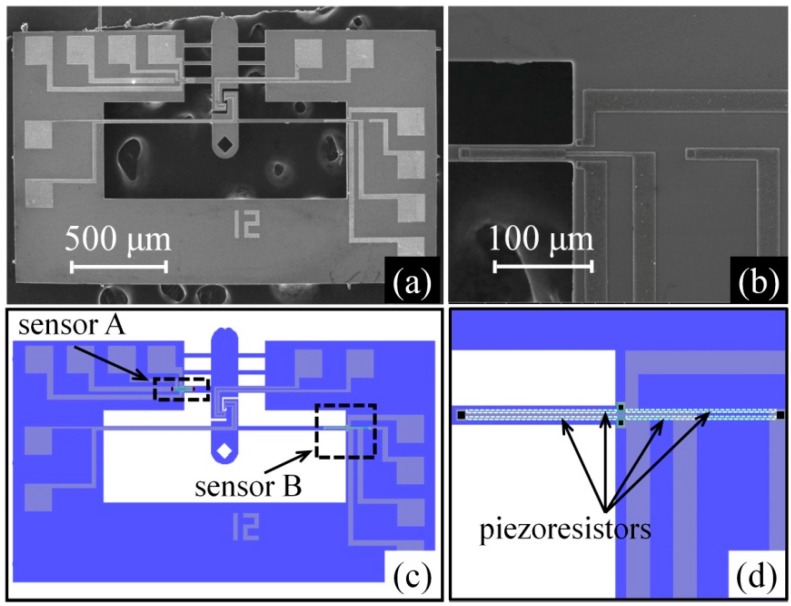
MEMS device with piezoresistive sensors: (**a**) SEM image of the device; (**b**) enlarged SEM image of the sensor B region; (**c**) lithography map of the device; and (**d**) lithography map of the sensor B region.

Using bulk silicon micromachining process, the MEMS device was fabricated on a 4 inch (100) SOI (Silicon-On-Insulator) wafer, consisting of a 380-μm-thick handle layer, a 60-μm-thick n-type device layer with resistivity of 1–2 Ω·m, and a 0.5-μm-thick buried oxide layer. The minimum feature size of the device structure is 6 μm. The device has an aspect ratio of ten. Figure 5 shows the main steps of the fabrication process. First, a 300-μm-thick SiO_2_ insulation layer was grown on each side of the wafer by thermal oxidation (Figure 5a). The thermal oxidation SiO_2_ layer on the device side was patterned and etched as a mask for subsequent ion implantation. The piezoresistors, with resistivity of $1.17\times 10^{-2}$ Ω·cm, were prepared by boron doping by means of ion implantation at 100 keV with a dose of $10^{15}$ ${\mathrm{cm}}^{-2}$ (p-doped, Figure 5b). Using the same process, electrical contacts were created with an implantation energy of 100 keV and a dose of $3\times 10^{15}$ ${\mathrm{cm}}^{-2}$ (p+ doped, Figure 5c). A 1-μm-thick aluminum film was then sputtered on the surface and then etched using potassium hydroxide (KOH) solution to form interconnects and pads (Figure 5d). The device layer was then etched by inductively coupled plasma (ICP) etching (Figure 5e). Finally, ICP was used to etch out the handle and buried oxide layers from the backside to create a movable structure (Figure 5f). Figure 6a shows a SEM image of a MEMS fabricated device. Figure 6b shows a magnified view of sensor B. The corresponding lithography maps are shown in Figure 6c,d. Comparison between the fabricated device and the lithography maps shows that the lateral undercutting of both ICP etching and aluminum film wet etching was less than 1 μm, indicating a well-controlled etching processes.

### 3.2. Device Calibration and Quantitative Tensile Testing

**Figure 7:**
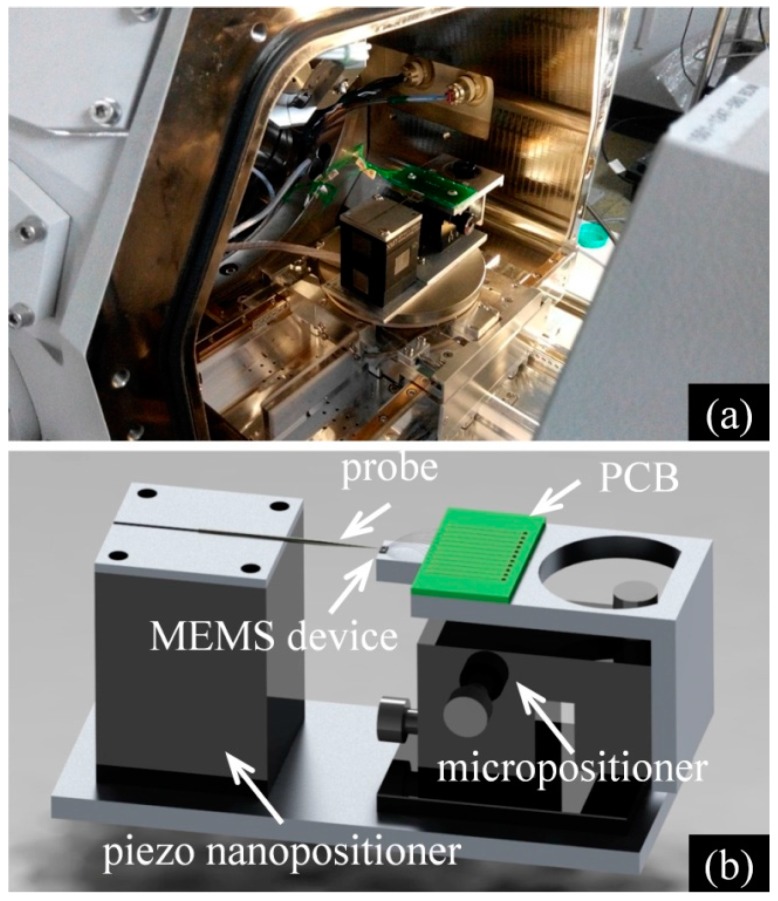
Setup for in situ tensile testing in SEM: (**a**) the optical image of the testing system equipped in SEM; and (**b**) schematic of the testing system.

A focused ion/electron dual-beam system (FIB/SEM, FEI Helios Nanolab 600 i, FEI, Hillsboro, USA) was used to calibrate the displacement of the MEMS device in a high vacuum environment ($10^{-5}$ Pa) with a purpose of avoiding the disturbances arising from variations in temperature, humidity, electromagnetic radiation, mechanical vibration, etc. [58,59], as shown in Figure 7a. Figure 7b shows the design of the testing system setup for calibration prior to tensile testing. The MEMS device was first glued on to a custom-made printed circuit board (PCB) and then connected with the PCB by ultrasonic bonding. The PCB was then fixed onto a three-dimensional micropositioner. The shuttle beam of the device was precisely aligned with the probe fixed on the piezo nanopositioner. The piezo nanopositioner moved in a step of 7 nm to drive the shuttle through the probe, ~10 μm in diameter. For each batch of MEMS devices fabricated on the same wafer, a few MEMS devices were selected for calibration. The displacements of the beams A and B on the selected calibration device were measured from the SEM images taken. The corresponding output voltages of the displacement sensors were simultaneously measured by a digital multimeter and recorded in a computer. A sensor voltage–image displacement curve is then obtained for each of the two sensors. During testing, the displacement on the sample is read out from the sensor voltage output of the two beams (*x*_a_ and *x*_b_) based on the calibration. The deformation strain is then calculated according to Equation (1) by knowing the original length (*l*_0_) of the specimen prior to testing. The force applied on the specimen *(F*_b_) is calculated based on the displacement of beam B, as per Equation (16). The stress can then be calculated using Equation (2) by measuring the cross-sectional area (*S*) of the specimen prior to tensile testing.

Using this testing system, uniaxial tensile tests were conducted on aluminum thin films in the FIB/SEM system. During the testing process, the piezocontroller, power suppliers, and multimeters, which were placed outside the FIB/SEM system, were connected with the device and the nanopositioner inside the system with shielded cables through the flange of the SEM.

### 3.3. Specimen Preparation

**Figure 8:**
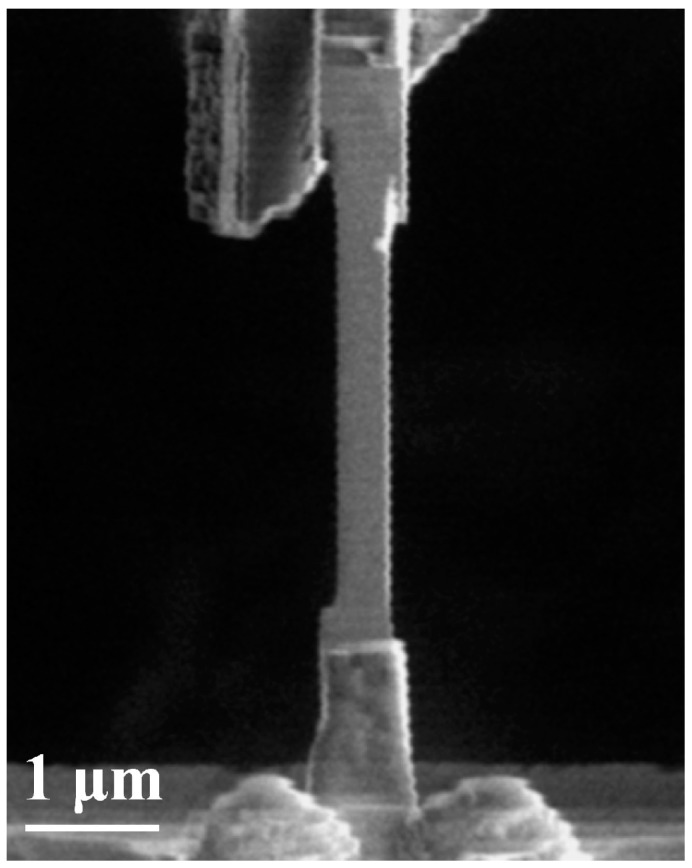
SEM image of a specimen fabricated for tensile testing.

The accuracy of the testing system was tested by measuring the stress–strain curve of aluminum film samples with different thicknesses. The aluminum films for tensile testing were prepared by DC magnetron sputtering. The purity of the aluminum target was 99.99% and the sputtering parameters included a working power of 400 W, vacuum level of $1.316\times 10^{-6}$ Pa and argon pressure of 2–3 Pa. An aluminum film with a thickness of ~1 μm was deposited on a thermally oxidized silicon wafer. Specimens for tensile testing in SEM were cut by FIB in the following sequence. First, a piece of the film was cut out with an ion beam using a current of 9.4 nA, lifted out and moved to the specimen stages on the MEMS device with a W probe, and fixed by Pt deposition on both ends. The film was then gradually thinned to hundreds of nanometers with the ion beam at a series of currents equal to 9.4 nA, 2.3 nA, 0.77 nA, and 7 pA to minimize the damage caused by the incident ion beam. A specimen with thicknesses of 510 nm is shown in Figure 8. The dimensions of the specimen are listed in Table 2.

## 4. Results and Discussion

### 4.1. Sensors Performance

**Figure 9:**
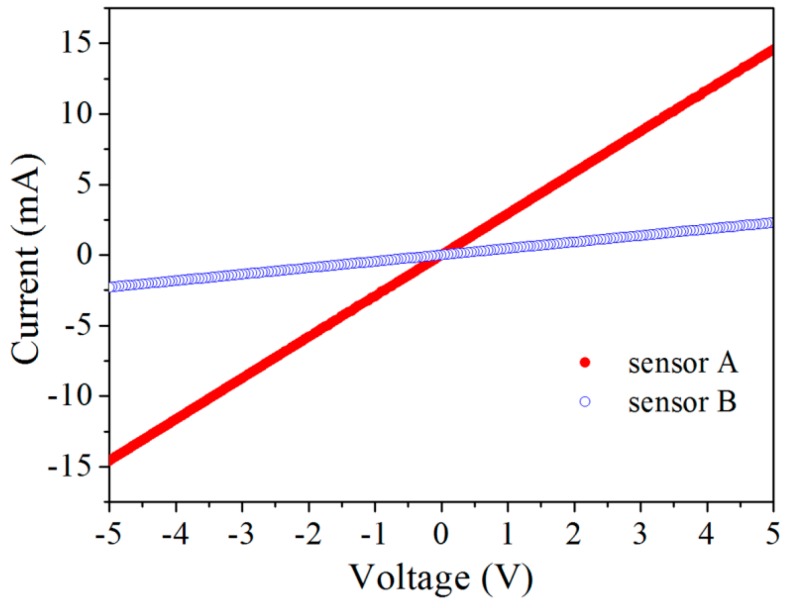
Current–voltage characteristics of the piezoresistors.

**Figure 10:**
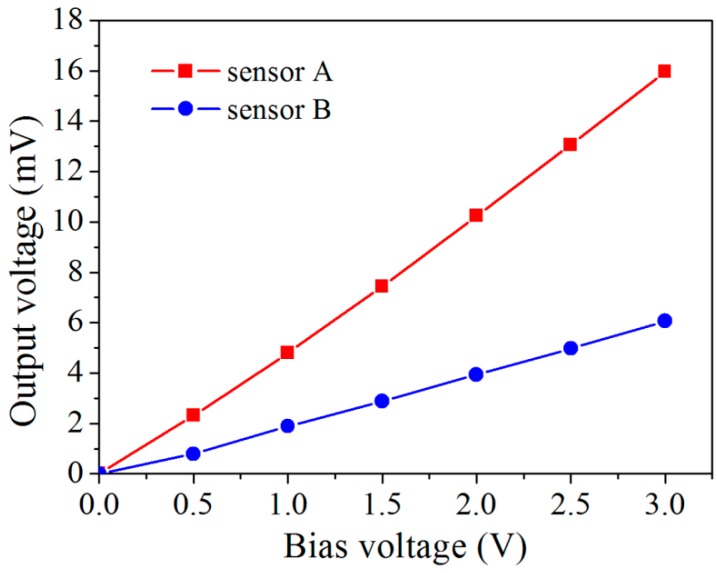
Zero-point deviation of the output voltage at different bias voltages.

Figure 9 shows the current–voltage characteristics of the two piezoresistive sensors. It is seen that both sensors exhibited perfect linear behavior within the range of −5–5 V. This also indicates a perfect contact between the sensors and the Al interconnects. The resistances of sensors A and B are determined to be 0.34 and 2.1 kΩ, respectively. Figure 10 shows the effect of the bias voltage on the zero-point output voltage of the two sensors. It is seen that the zero-point output also has a linear dependence on the bias voltage for both sensors. The zero-point deviation can be attributed to the uneven ion implantation among the four resistors, despite identical processing conditions used. Therefore, zero setting of the bridge output voltage was conducted on each sensor before calibration.

**Figure 11:**
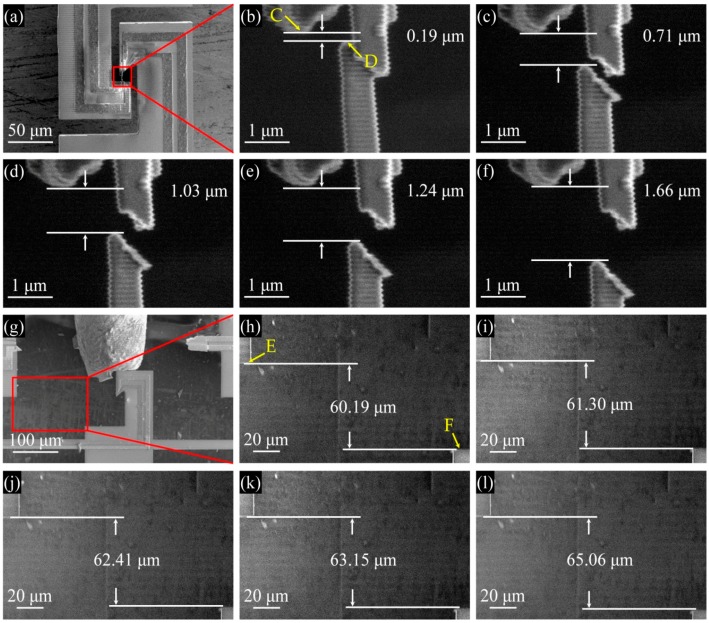
Displacement calibrations of sensors in SEM: (**a**–**f**) the displacements of the two reference points for sensor A; and (**g**–**l**) their displacements for sensor B.

**Figure 12:**
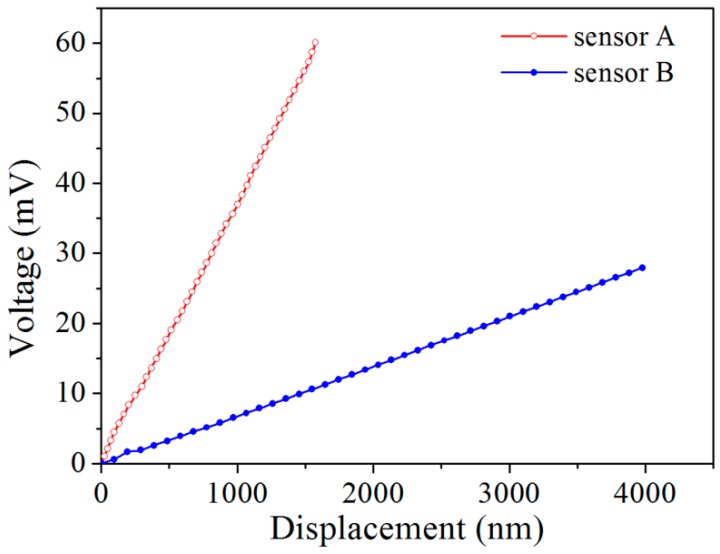
Output voltage as a function of the displacement.

Figure 11 shows SEM images used to calibrate the displacements measured by the sensors A and B. For each sensor, the beam center deflection, on which the sensor is located, was obtained by measuring the distance variation between two reference points in SEM, i.e., points C and D for sensor A (Figure 11a–f), and E and F for sensor B (Figure 11g–l). The movements of beams A and B were driven by the piezo nanopositioner through the shuttle beam and probe with a movement step size of 7 nm. Beams A and B need to be calibrated separately. For this, beam A was calibrated prior to beam B until rupture. When beam B was calibrated, beam A was already in the broken state as shown in Figure 11g. Figure 12 shows the output voltages of the two sensors versus the measured beam center deflections under a bias voltage of 3 V. It is seen that the sensor outputs are practically linear against the beam deflection. Using linear fitting, the displacement sensitivity is determined to be 37.4 μV/nm for sensor A and 4.8 μV/nm for sensor B. Comparing with the theoretical values listed in Table 1, the deviations are 51.5% and 17.9% for the two sensors, respectively. This is commonly attributed to the non-uniformity of ion implantation and excess resistance in the bridge [60]. The linearities of the sensors A and B are calculated to be 1.92% and 1.94%, respectively. Both values are smaller than the standard of a good linearity, i.e., 2%, demonstrating good sensor performance.

The maximum displacement of the beam center achieved in this calibration is <5 μm. This is far smaller than the beam length, thus the deflection can be treated as a linear system and described by the small deflection theory [61]. Therefore, we take the stress defined by Equation (16) as the load applied on the clamped beam. Based on the displacement sensitivities of the sensors A and B and Equation (10), the corresponding displacement resolutions are determined to be ~0.19 nm for sensor A and ~6.8 nm for sensor B. The sample elongation resolution is then determined to be 6.8 nm. The force resolution of sample/sensor B is then calculated to be 2.1 μN using Equation (16). Since the displacements of the sensors are measured by imaging method, the resolution that can be experimentally determined depends on that of the SEM or TEM used. Further work on TEM is required to determine more accurately, the displacement resolution.

It is well known that the electron beam inside an electron microscope can lead to electron accumulation in the device, which may interfere with the sensor behavior. To assess the influence of the electron beam on the sensor displacement sensitivity, sensors A and B were irradiated under the electron beam at an accelerating voltage of 2 kV (operating voltage) inside a SEM under magnifications of 2000×, 8000× and 16,000×. Table 3 shows the sensitivities of the sensors under irradiation of e-beam in SEM. The results show that no apparent changes of sensitivity can be detected, indicating a neglected influence of electron beam on sensor sensitivity.

### 4.2. Stress–Strain Curve

**Figure 13:**
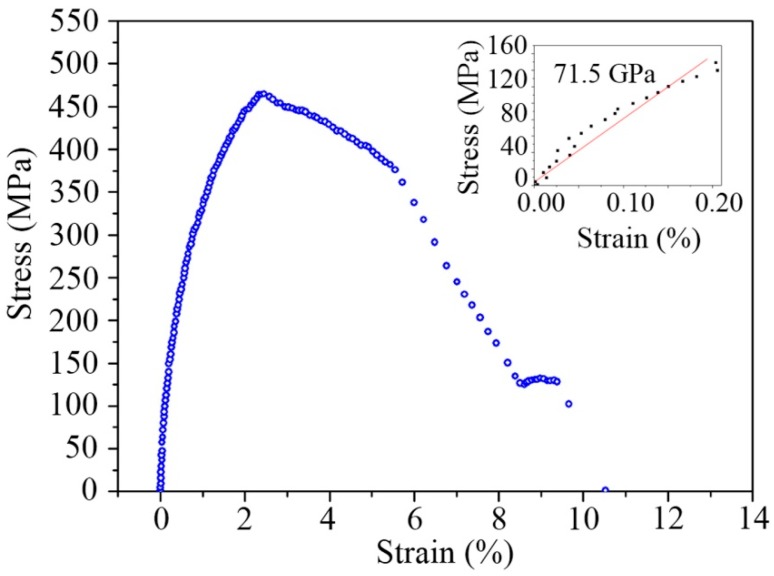
Stress–strain curve of an Al film with thickness of 510 nm.

An Al thin film of 510 nm in thickness was subjected to tensile deformation on the device in the FIB/SEM system with an actuation step size of 7 nm. SEM images were taken during tensile deformation. Figure 13 shows the stress–strain curve measurement of the Al film sample. The Young’s modulus of the specimen is measured to be 71.5 GPa by linear fitting, consistent with bulk materials (68.5–71 GPa). The Young’s modulus is also consistent with the 100 nm Al film measured by the MEMS sensors [62]. The flow stress is determined to be ~460 MPa, which is low compared to ~700 MPa [62]. This may be related to the thickness and density of the film used.

## 5. Conclusions

A small sized MEMS device was designed and fabricated to study quantitatively, the mechanical properties of thin film materials in situ during TEM/SEM observation. The device was tested in SEM. Piezoresistive sensors were integrated onto the device to measure quantitatively the mechanical properties of materials. This device allows the study of microstructure–property correlations in small materials and understanding of deformation mechanisms at nanometric and atomic scales. The main outcome of this work may be summarized as follows:

(1) Sensors A and B have displacement sensitivities of 37.4 μV/nm and 4.8 μV/nm.
(2) Sensor A has a theoretical displacement resolution of 0.19 nm and sensor B has a force resolution of 2.1 μN.
(3) The MEMS device has a displacement range limit of 5.47 μm and a theoretical load range limit of 55.0 mN.
(4) Measurement of the Young’s modulus of the Al film by the device verifies the reliability of the sensors.
(5) The device has a dimension small enough to be integrated on the TEM holder to study the property–structure correlation at the atomic scale.

## Figures and Tables

**Table 1:** Parameters of the two piezoresistive sensors.

Parameters	Value		Unit
	Sensor A	Sensor B	
*Α*	10^−5^		-
*T*	300		K
μ_p_	0.934 × 10^−2^		cm^2^·V^−1^·s^−1^
Bias voltage	3.0		V
*f*_max_	1000		Hz
*f*_min_	10		Hz
Clamped beam length	150	600	Μm
Clamped beam width	60	60	Μm
Clamped beam thickness	30	15	Μm
Piezoresistor length	46	107	Μm
Piezoresistor width	8	3	Μm
Piezoresistor thickness	1.1	1.1	Μm
Theoretical displacement sensitivity	77.1	7.1	μV/nm
Theoretical displacement resolution	0.19	4.6	nm
Theoretical displacement range limit	5.47	175.14	μm
Theoretical load range limit	888	55.0	mN

**Table 2:** Dimensions of the Al specimen.

Length (nm)	Width (nm)	Depth (nm)
4880	620	510

**Table 3:** Sensitivity of the two sensors under irradiation of e-beams.

Sensors	Magnification		
	2000×	8000×	16,000×
Sensor A	37.5 μV/nm	37.2 μV/nm	37.4 μV/nm
Sensor B	4.6 μV/nm	4.9 μV/nm	4.8 μV/nm
